# Delayed Onset Bilateral Vocal Cord Palsy in Miller Fisher Syndrome: The Rehabilitation Outcome

**DOI:** 10.7759/cureus.65799

**Published:** 2024-07-30

**Authors:** Nicole H Chen, Abbas Bin Zainul Abideen, Tze Chao Wee

**Affiliations:** 1 Rehabilitation Medicine, Changi General Hospital, Singapore, SGP

**Keywords:** case report, robot assisted gait training, rehabilitation, bilateral vocal cord paralysis, miller fisher syndrome

## Abstract

Miller Fisher syndrome (MFS) typically presents with acute development of ataxia, ophthalmoplegia, and areflexia. Bilateral vocal cord palsy (BVCP) is a rare manifestation of MFS. We present a case of a 66-year-old male diagnosed with MFS complicated by an unusually delayed onset of BVCP while undergoing inpatient rehabilitation. We also describe the inpatient rehabilitation course, including the use of a patient-guided suspension system (PGSS) as a therapeutic adjunct to aid gait training, resulting in significant functional improvement in ambulation and activities of daily living. Given the rarity of BVCP in MFS, this case highlights the importance of healthcare professionals being aware of this phenomenon so that prompt treatment can be initiated to reduce significant morbidity. Innovative treatment approaches such as the use of a PGSS may also prove beneficial in the rehabilitation of patients with MFS with significant ataxia.

## Introduction

Miller Fisher syndrome (MFS) is a rare variant of Guillain-Barré syndrome (GBS), an acute immune-mediated polyneuropathy. Worldwide incidence of GBS is 1-2 per 100,000. MFS accounts for 1-7% of GBS cases in the West but in Asia, it accounts for up to 15-20% of GBS cases [1]. The clinical hallmark of MFS is the symptomatologic triad of acute ophthalmoplegia, ataxia, and areflexia in the setting of a preceding bacterial or viral illness. While the involvement of cranial nerves, in particular the facial nerve, has previously been reported, bilateral vocal cord palsy (BVCP) is a rare manifestation of MFS with only four accessible case reports available [2]. Past research supports early and intensive multidisciplinary rehabilitation for patients with GBS to promote functional recovery [3]. One high-quality randomized controlled trial demonstrated the effectiveness of higher-intensity multidisciplinary ambulatory rehabilitation in reducing disability in persons with GBS in the later stages of recovery, compared with lesser-intensity rehabilitation intervention for up to 12 months. Four observational studies, further demonstrated some support for improved disability and quality of life following inpatient multidisciplinary rehabilitation up to 12 months [3]. However, there are no established guidelines on the rehabilitation of MFS. Here, we present a case of delayed BVCP in MFS to underscore the importance of vigilance for this complication. This case also serves to highlight the use of a patient-guided suspension system (PGSS) as a therapeutic adjunct, a novel approach in the rehabilitation of a patient with MFS.

## Case presentation

A 66-year-old male presented with giddiness and blurred vision upon waking up. This was preceded by three days of cough and sore throat. He had a past medical history of well-controlled hypertension, hyperlipidemia, and impaired fasting glucose. The patient was afebrile and his vitals were stable, and within normal limits. On initial examination on presentation to the hospital, he had bilateral complete ophthalmoplegia. Medical Research Council (MRC) grading was 5 in all four limbs although movement was ataxic. Biceps, brachioradialis, knee, and ankle reflexes were absent throughout. Over the next 24 hours, he developed dysphagia and bilateral ptosis.

Laboratory tests revealed normal complete blood count, and renal and liver profiles. Magnetic resonance imaging (MRI) scan of the brain showed no acute infarct or intracranial hemorrhage. The neurology team was consulted, and a lumbar puncture was done on day two of admission. This did not reveal cytoalbuminologic dissociation (Table 1). Upon further testing, serum anti-GQ1b antibody was detected as being strongly positive. No nerve conduction study was performed as diagnostic clarity had been achieved. The diagnosis of MFS was made on the basis of clinical presentation and supporting laboratory findings.

**Table 1:** Results of lumbar puncture RBC: red blood cell; CSF: cerebrospinal fluid; CNS: central nervous system; AFB: acid-fast bacilli

Lumbar puncture	Result	Reference range
Appearance	Clear and colorless	-
Cell count	2 cells/mm^3^	-
Red blood cell (RBC) count	Not detected	-
Protein (total)	0.31 g/L	0.10-0.40 g/L
Glucose	4.0 mmol/L	2.4-5.5 mmol/L
Gram stain smear	Negative	-
CSF culture	No bacterial growth	-
CNS culture (anaerobes)	No growth of anaerobes	-
AFB smear	Negative	-
Meningitis multiplex assay	No viral, bacterial, or fungal pathogens detected	-

On day three of admission, the patient was commenced on intravenous immunoglobulin 2 g/kg over five days. Nasogastric tube was inserted on day four due to worsening dysphagia. Otherwise, he remained clinically stable with no need for oxygen supplementation.

The patient was transferred to the inpatient rehabilitation ward on day 10. On day 16, he developed stridor and oxygen saturation dropped to 88% on room air. Urgent nasendoscopy showed BVCP. He was intubated and transferred to the intensive care unit (ICU) and eventually underwent tracheostomy on day 21. The patient had increased respiratory secretions requiring frequent suctioning. He was therefore only transferred back to the inpatient rehabilitation ward on day 49 when suctioning requirements were reduced.

He required maximal assistance for transfers and had poor sitting balance due to severe trunk ataxia. He required maximal assistance in performing his activities of daily living (ADL). Initially, the patient participated in conventional physiotherapy. This included bed mobility and sitting balance exercises, progressing to sit-to-stand exercises (five to 10 repetitions), and marching on the spot (five sets of four repetitions). At the two-week mark, he was still unable to proceed with overground ambulation even with a platform rollator, due to severe ataxia of all four limbs and extreme fear of falling.

Due to the inability to progress with gait training using conventional methods, our team decided to commence therapy on a PGSS (Andago; Volketswil, Switzerland: Hocoma) as a therapeutic adjunct. Our team believed that the PGSS would be able to provide the intensive gait therapy our patient needed while maintaining safety with the use of supportive harnesses. Furthermore, as the PGSS allows dynamic and adjustable weight support, it enables lateral weight shift and hence would provide balance practice for the patient. The PGSS also allows free overground walking, where the patient can practice walking on different terrains.

During the first session on the PGSS, the patient was able to walk 225 m with two rest stops in 30 min. He was greatly motivated and his fear of falling was also much reduced. He completed seven sessions with 5 kg weight support on either side as detailed in Table 2 and transited to a conventional overground gait training program. The patient was able to ambulate with a rollator frame for 30 m, with minimal assistance from the therapist. At this point, he still had excessive sway at the hips, with occasional suboptimal foot placement due to ataxia. However with further physiotherapy, his balance and gait pattern improved and he was able to walk up to 180 m with supervision, without aid on level ground.

**Table 2:** Details of patient-guided suspension system (PGSS) therapy.

Session number	Day of admission	Time (min)	Distance (m)
1	63	30	225
2	64	30	300
3	65	30	222
4	66	30	285
5	69	30	372
6	70	30	375
7	71	30	433

Respiratory training included diaphragmatic breathing exercises and inspiratory muscle strengthening. He was taught the Active Cycle of Breathing Technique (ACBT) to mobilize and clear excess pulmonary secretions. The patient underwent intensive swallow rehabilitation comprising oral motor exercises including jaw strengthening exercises, tongue strengthening exercises (pushing the tongue against resistance from a tongue depressor), and lip closure exercises. He then progressed to effortful swallow exercises for pharyngeal strengthening and also started on thermal tactile oral stimulation with a cold spoon to elicit swallows. The patient commenced oral trials with up to five teaspoons of honey-thick fluids, three times a day. This progressed to 50 mL of honey-thick fluids via teaspoon three times a day, which he tolerated well. On day 69, fiberoptic endoscopic evaluation of swallowing (FEES) was performed. This revealed moderate to severe oropharyngeal dysphagia characterized by reduced orolingual control and pharyngeal contraction. Mild penetration was observed on mildly thick fluids but not on extremely thick fluids. As no aspiration was observed, he was deemed safe for oral feeding, with accepted penetration risks. A nasogastric tube was removed and he tolerated a pureed diet with honey-thick fluids.

A repeat nasoendoscopy on day 93 revealed his vocal cords were mobile. He was started on a trial of spigotting as per our hospital protocol and was successfully decannulated on day 99. Voice therapy involved vocal exercises and vocal hygiene education, together with speech drills to improve articulatory precision. 

Occupational therapy intervention initially focused on the management of the patient’s ataxia. This included sensory re-education, modified open and closed chain tasks, and weight-shifting practice. Subsequently, bilateral upper limb and ADL retraining were carried out.

Prior to transferring to a community hospital on day 106, the patient could ambulate with supervision, without aid 180 m on level ground. He required standby assistance in performing his ADL. He tolerated an easy chew diet and thin fluids without complications, and speech intelligibility was approximately 80%.

The patient made remarkable improvements in his Functional Independence Measure (FIM) scores. His total FIM score on admission to the rehabilitation unit was 24. Upon discharge, the total FIM was 102, representing impressive total FIM gains of 78. Motor FIM scores increased from 13 to 67. Similarly, Modified Barthel Index scores improved from 0 to 79, reflecting increased independence in ADL.

## Discussion

BVCP is a rare manifestation of MFS. A review of the literature identified four accessible case reports (Table 3) [4-7]. All cases presented with signs of acute respiratory distress fairly early upon onset of symptoms, ranging from two to 11 days. Our case, however, developed stridor and acute desaturation on day 16, demonstrating a delayed onset of BVCP. Therefore, it is imperative that healthcare professionals remain vigilant of BVCP being a complication of MFS even if the patient shows clinical improvement.

**Table 3:** Case reports of BVCP in MFS and their clinical features. BVCP: bilateral vocal cord paralysis; GBS: Guillain-Barré syndrome; MFS: Miller Fisher syndrome; ABPp: acute bulbar palsy plus syndrome

Studies	Demographics	Initial symptoms	Onset of BVCP (days since initial symptom onset)	Diagnosis	Recovery of BVCP	Anti-GQ1b antibody
Chavada et al. 2022 [4]	76 years old male	Difficulty swallowing/speaking drooping of eyelids	Day 2	ABPp	Yes, de-cannulated after 4 months	Negative
Ramakrishna et al. 2020 [5]	76 years old male	Unsteady gait poor oral intake dysarthria dizziness	Day 7	MFS	No, tracheostomy- dependent at least 6 months	Negative
Frentescu et al. 2020 [6]	60 years old male	Vomiting, dysphonia hypertension, vertigo, and unsteady gait	Day 2	MFS	Yes, de-cannulated after 3 months	Positive
Bahk et al. 2021 [7]	65 years old male	Dizziness diplopia	Day 11	MFS/GBS overlap syndrome	No, tracheostomy- dependent at least 7 months	Unclear

A tracheostomy was required in all cases cited above in Table 3 including ours, to secure the airway. Recovery of BVCP with successful decannulation occurred within three to four months for two out of four reported cases while the other two patients remained tracheostomy-dependent even by six to seven months. The use of posterior cricoarytenoid (PCA) reinnervation and pacing was previously described in a case report, offering promise for amelioration of respiratory compromise in a patient with MFS and BVCP [8]. There have been no further reports of such intervention in MFS since.

This study also demonstrates the use of PGSS as a therapeutic adjunct in the rehabilitation of a patient with MFS who was unable to participate in conventional rehabilitation gait training because of severe ataxia. Andago is a patient-directed dynamic body-weight supported suspension system that allows over-ground walking at a patient-determined speed. Sensors and control algorithms assist the device with forward propulsion, maneuvering of the frame, and directional adjustments for patient safety. Other safety features of the device include collision-sensing auto brakes that activate on minimal impact and harnesses that prevent the patient from falling. Although robot-assisted gait training has been described in the rehabilitation of patients with stroke-related ataxia, the use of PGSS in the rehabilitation of a patient with MFS has not been previously reported in the literature to the best of our knowledge [9]. The use of the PGSS enabled our patient to have increased training duration and distance while reducing the therapist’s energy expenditure. By providing repetitive and task-specific gait training, PGSS helps improve gait patterns and functional mobility. Additionally, the adjustable and asymmetrical weight support provided allows for proper lateral weight shift, which is crucial in balance training. All these facilitated a rapid transition to an overground gait training program and a remarkable improvement in the patient’s functional mobility as evidenced by improved motor FIM scores.

## Conclusions

This study highlights a case of delayed presentation of BVCP in MFS. Despite BVCP being a rare manifestation of MFS, healthcare professionals should be aware of this phenomenon when providing care for patients with MFS. This ensures that prompt emergency treatment can be delivered to patients who develop acute respiratory compromise from BVCP and reduces significant morbidity. Innovative treatment approaches such as PGSS robotic devices that are traditionally used in stroke patients may also prove beneficial as a therapeutic adjunct to conventional physical therapy in the rehabilitation of patients with MFS. Further research is warranted to elucidate optimal rehabilitation strategies and protocols in this population.

## References

[1] Noioso CM, Bevilacqua L, Acerra GM (2023). Miller Fisher syndrome: an updated narrative review. Front Neurol.

[2] Berlit P, Rakicky J (1992). The Miller Fisher syndrome. Review of the literature. J Clin Neuroophthalmol.

[3] Khan F, Amatya B (2012). Rehabilitation interventions in patients with acute demyelinating inflammatory polyneuropathy: a systematic review. Eur J Phys Rehabil Med.

[4] Chavada IJ, Thampi SJ, Babu D, Natarajan V (2022). Guillain-Barré syndrome without limb weakness: a rare variant with acute bulbar palsy. J Family Med Prim Care.

[5] Ramakrishna KN, Tambe V, Kattamanchi A, Dhamoon AS (2020). Miller Fisher syndrome with bilateral vocal cord paralysis: a case report. J Med Case Rep.

[6] Frentescu A, Feys O, Mangion JP, Osseman M, Horlait G (2020). Miller Fisher syndrome associated with respiratory failure. Int J Case Rep.

[7] Bahk J, Yang W, Fishman J (2021). Bilateral vocal cord paralysis in Miller Fisher syndrome/Guillain-Barré overlap syndrome and a review of previous case series. BMJ Case Rep.

[8] Broniatowski M, Grundfest-Broniatowski S, Hadley AJ (2010). Improvement of respiratory compromise through abductor reinnervation and pacing in a patient with bilateral vocal fold impairment. Laryngoscope.

[9] Belas Dos Santos M, Barros de Oliveira C, Dos Santos A, Garabello Pires C, Dylewski V, Arida RM (2018). A comparative study of conventional physiotherapy versus robot-assisted gait training associated to physiotherapy in individuals with ataxia after stroke. Behav Neurol.

